# Multiple Unit Particles System of Ramipril: An Approach to Enhance Stability

**DOI:** 10.4103/0975-1483.80291

**Published:** 2011

**Authors:** HP Patel, JK Patel, MP Patel, RR Patel

**Affiliations:** *Torrent Research Centre, Gandhinagar, Gujarat, India*; 1 *Nootan Pharmacy College, S.P. Sahkar Vidhyadham, Visnagar - 384 315, Gujarat, India*

**Keywords:** Colloidal silicone dioxide, microcrystalline cellulose, ramipril

## Abstract

The present invention relates to produce multiple unit particle system (MUPS) of stabilized ramipril pellets, hydrochlorothiazide, diluents, superdisintegrants, and lubricants which produce better dissolution of the system for better bioavailability with improving stability and bioavailability of ramipril. More particularly, the present invention is directed for stabilized ramipril against decomposition into degradation products, namely ramipril-DKP and ramipril-diacid, during formulation and storage conditions. Simple ramipril formulation shows 15.15% related impurities after 3-month accelerated stability study, which was minimized to the 2.07% related impurities in ramipril pellets after 6 month accelerated stability study. By making MUPS of ramipril pellets, Hydrochlorothiazide and other excipients show better dissolution (100.4% of ramipril and 97.9% of hydrochlorothiazide within 60 min) to produce better bioavailability. So, making MUPS containing ramipril pellets with polymer coating and hydrochlorothiazide and other excipients shows better stability of ramipril along degradation and synergistic effect among hypertension in immediate delivery.

## INTRODUCTION

Conventional immediate release drug delivery system is based on single- or multiple-unit reservoir or matrix system, which are designed to provide immediate drug levels in a short period of time. Immediate release drug delivery is desirable for drugs having long biological half-life, high bioavailability, lower clearance, and lower elimination half-life. But main criterion for immediate release dosage form is poor solubility of the drug and its immediate action to treat any unwanted defect or disease. Hypertension, commonly referred to as “high blood pressure,” is a medical condition where the pressure is chronically elevated; it is one of the commonly found diseases, affecting most of the populations in the world. So, treating hypertension effectively is the main criterion of this study. For treating hypertension, commonly used drugs include ACE inhibitors, alpha blockers, beta blockers, calcium channel blocker, diuretics, and combination of any of these categories if immediate action is required.

Conventional immediate release drug delivery system is based on single or multiple-unit reservoir or matrix system, which are designed to provide immediate drug levels in short period of time. Immediate release drug delivery is desirable for drugs having long biological half-life, high bioavailability, lower clearance, and lower elimination half-life. But main criterion for immediate release dosage form is poor solubility of the drug and its immediate action to treat any unwanted defect or disease.

Ramipril is a prodrug and is converted to the active metabolite ramiprilat by liver esterase enzymes, and is an angiotensin-converting enzyme (ACE) inhibitor, used to treat hypertension and congestive heart failure. Its long biological half-life (3-16 h), its dose (2.5 mg/day), and long elimination phase (9-18 h) suggest its immediate action for treating hypertension.[1] Studied combination of ramipril with other diuretic provides synergistic antihypertensive effect in controlling blood pressure in hypertension along with a significant regression of left ventricular hypertrophy. Also, hypertensive patients, who did not show much response to monotherapy with ramipril, showed significant reduction in blood pressure when shifted to ramipril-diuretic combination. Among all diuretics, hydrochlorothiazide has the low dosage, poor solubility, and long half-life to treat hypertension. So, combination of ramipril and hydrochlorothiazide provides synergistic effects in the treatment of mild to moderate hypertension.[2] So, this study focused on the development of immediate drug delivery of ramipril and hydrochlorothiazide. Ramipril needs special care when formulating into pharmaceutical preparations due to the physical stress associated with formulating processes which can increase the rate the decomposition of ramipril into degradant products. Indeed, factors that influence the stability of ramipril formulations are mechanical stress, compression, manufacturing processes, excipients, storage conditions, heat, and moisture.[3–5] So, the special formulation for ramipril is pellets, which gives more stability to ramipril from compression and other stress condition during formulation and storage conditions. And also pellets offer some additional advantages like dispersing freely in GI tract, maximizing drug absorption, and minimizing local irritation of the drug, which indicates that pellets can be used for immediate drug delivery.[6,7]

With regard to the final dosage form, the multiparticulates can be filled into hard gelatin capsules or compressed into tablets. The compression of multiparticulates into tablets is becoming more popular, especially in the USA, where hard gelatin capsules have been tampered.[8–10] So, this study focused on the development of immediate release tablets containing pellets.

## MATERIALS AND METHODS

### Materials

Ramipril (Hetero labs), hydrochlorothiazide (Unichem labs), microcrystalline cellulose (FMC Biopolymer), mannitol (Roquette, Signet Chem. Corp), starch (Roquette), cross-carmellose sodium (FMC Biopolymer), hydroxy propyl methyl cellulose (Shinetsu), talc (Ferro-Belgium), magnesium stearate (Ferro-Belgium), colloidal silicone dioxide (Degussa).

### Preliminary trials

Preliminary study trials were carried out for the formulation of ramipril /HCTZ tablets. Literature survey shows that ramipril is very sensitive to light moisture, physical or chemical stress, and in contact with this ramipril is degrade in to the ramipril-diketopiperazine by cyclization or by condensation.[11–13] This is tested by doing these preliminary trials for tablet formulation. For the stability improvement and better formulation of ramipril and hydrochlorothiazide, preliminary trials by direct compression method and wet granulation method were tried and compacted in to tablets. And bilayer technology of both individual drugs was also tried for better formulation by compaction method.

### Preparation of ramipril pellets by fluidized bed processing method

Trial and error method was used for the development of formulation of ramipril pellets. With reservoir-type coated pellet dosage forms, the polymeric coating must be able to withstand the compression force; it can deform but should not rupture. Without sufficient elasticity of the film, the coating could rupture during compression and the extended release properties would be lost. In addition, the bead core should also have some degree of plasticity, which can accommodate changes in shape and deformation during tableting.[14] So, MCC can be used for the core material for drug coating. But direct drug coating on MCC will create compatibility problem or stress to the ramipril; so, film coating on MCC will overcome this problem and give the elasticity to the drug as well. Film-coated ramipril pellets were prepared by seal coating with hydroxy propyl methyl cellulose and then drug coating on same pellets and finally film coating was performed on drug-coated pellets.

### Seal coating on base materials

Polymer used for seal coating was hydroxy propyl methyl cellulose, which is used as film former in 5–15% concentration. So, trial started with 5% concentration of polymer for seal coating. And glidant was used in min concentration and seal coating was done on MCC as a core material.[15] Seal coating on MCC was done by fluid bed processor (GLATT machine) using solution of hydroxy propyl methyl cellulose in water with talc as a glidant.

### Drug coating on seal-coated pellets

Drug coating was performed on seal-coated pellets along with binder hydroxy propyl methyl cellulose. Polymer concentration had been taken from 2% to 6% because hydroxy propyl methyl cellulose has been used as binder in this range of concentration. Here binder was used because drug particles can stick to the seal-coated pellets and make a uniform drug coating on seal-coated pellets and appropriate amount of drug to be contained in selected quantity of pellets. Drug coating on seal-coated pellets was done by the solution of ramipril in solvent with binder in fluid bed processor.

### Film coating on drug-coated pellets

Film coating on drug-coated pellets was varied by different concentration of hydroxy propyl methyl cellulose polymer used as a film former. For film coating, polymer concentration taken is in the range of 5-15%. Theoretically amount of polymer required for film coating is higher than seal coating. So, for film coating, polymer concentration taken was in the range of 5-15%. Talc was added for reducing the static charge into pellets. By adding talc into spraying solution, it helps evaporate during spraying, stick to the pellets, and remove the static charge of pellets during spraying and drying.[16]

### Tableting of coated pellets with hydrochlorothiazide and other excipients

Ramipril/hydrochlorothiazide tablets were done by using different concentration of and different proportion of diluents. Disintegrating agent like sodium starch glycolate (SSG) was used in different concentration in tablet formulation. The usual concentration of SSG employed in a formulation is between 2% and 8%,[17,18] with the optimum concentration of about 4%, although in many cases 2% is sufficient.[19–21] All disintegrating agents are water insoluble. Disintegrant is added to the formulations to facilitate breakup of the tablet when in contact with water in the GI tract. They may function by drawing water into the tablet and swelling causing it to burst apart.[22,23]

Diluents like MCC and mannitol were used for formulation development. Because, tableting of pellets with diluents produce breakage or rupture of pellets and that will affect the dissolution profile and stability of tablet. This problem is solved by using different granular MCC grade, which are available with greater plasticity.[24,25] So, granular grade of MCC and mannitol were used for the trial of tablets.

### Evaluation properties of pellets[26–28]

Particle size distribution study of pellets was performed by sieving analysis using a nest of standard sieves and the desirable range of pellets was taken to be between 450 and 1180 mm. The post-compaction tests of pellets were performed; they included assay, *in-vitro* dissolution study in 0.1 N HCl at specified time interval, and measuring the concentration release in time profile by HPLC. Ramipril pellets were subjected to the accelerated stability studies in aluminum/aluminum pouch. As the dosage form is formulated to deliver it into stomach, no change should occur in its % dissolution profile and related impurities. Ramipril is very sensitive to light, moisture, and any physical or chemical stress. For this study, tablets prepared from pellets and only pellets were packed in aluminum pouch and in vial, respectively, charged for accelerated stability study at 40°C and 75% RH for 3 months in a chamber. Stability study of pellets was performed by first checking the initial parameters of pellets and then put it in specified condition for 1 month. After 1 month, check for all parameters. If it shows satisfactory results, then continue the test for next month and continue for 3 months.

### Evaluation properties of tablets

The post-compaction tests were performed on the tablets at least 24 h following preparation and they included the determination of weight, thickness, drug content uniformity, friability (Erweka friabilator, Germany), hardness tablet hardness tester, Schleuniger Pharmaton 6D Model, USA, disintegration (Erweka ZT 44, Germany), and dissolution testing in 0.1 N HCl, for formulations containing coated pellets.[29–31] All tests were performed on at least five replicates. The degree of damage to drug pellets was assessed by the use of the *similarity factor*…

*F*_2_= 50 * log {[1 + (1/*n*) ∑ _t=1_^n^(R*_t_*— T*_t_*)^2^] ^- 0.5^* 100},

where R*_t_* and T*_t_* are the percent dissolved at each time point for reference (R) and test (T) products. An F_2_ value between 50±100 suggests that the two dissolution profiles are similar and the mean dissolution profiles are assumed to differ by no more than 15% at any time point.[32] An accelerated stability study was also performed in aluminum/aluminum pouch of the tablets.

## RESULTS AND DISCUSSION

### Preliminary trials

Literature survey showed that ramipril is very sensitive to light moisture, physical, or chemical stress, and in contact with this ramipril is degraded into the ramipril-diketopiperazine by cyclization or condensation. This is tested by doing these preliminary trials for tablet formulation. In the direct compression flow of the blend and parameters of tablets were major problem. Where in wet granulation method, parameters are in range but as the properties of ramipril, it is degraded and showing stability problem [Table 1]. Same problem also can be there in bilayer formulation because it was also prepared by stress. Also the tablet parameters were not within the range.

**Table 1:** Accelerated stability study data of tablets prepared by wet granulation method

Parameters	40°C 75%RH 1M	30°C 75%RH 1M
% Assay (ramipril:HCTZ)	93.3/95.5	99.3/96.2
% Related impurities (total impurity)	10.2	6.82
% Dissolution (ramipril:HCTZ)	90.3/90.3	95.1/90.4

### Seal coating on microcrystalline cellulose

By using granular grade of MCC, pellets were in spherical shape and properly form. From the comparisons of sieving analysis of different concentration showed that in 7% polymer film coating, pellets are formed and it is very regularly distributed in 60# sieves than other two concentrations. Pellets of this batch were not breaking easily which showed good film formation than other two batches. In 5% film coating, fines of powder were seen to be very high than other two batches; the small quantity of pellets formation was also seen here than the other two batches. The pellets were breaking freely which means the film coating was not properly done. In 9% film coating, aggregation of pellets was seen and pellets were sticking to each other and somewhat slugging observed. So, for next experiment of drug coating on polymer-coated pellets will be done with 7% polymer seal-coated pellets. Sieve analysis data of different batches are shown in Table 2.

**Table 2:** Sieve analysis results of different % concentrations of polymer

ASTM sieve no.	%Weight retained of pellets		
	5%Polymer	7%Polymer	9%Polymer
20	0	0	0
40	0.32	12.2	21.6
60	53.6	74.9	72.1
80	23.7	6.3	4.1
100	15.2	2.4	0.9
Below 100	7.18	4.2	1.3

### Drug coating on seal-coated pellets

Drug coating on the seal-coated pellets with different proportion of binder concentration was evaluated. Three different concentrations of binder (2%, 4%, 6%) were taken for drug coating. But by appearance of all three batch pellets, 4% binder concentration pellets were seen uniform in shape and size. Sieve analysis study of all three batches showed that drug-coated pellets were retained highest on 40# sieve 4% binder concentration than other two concentrations. And also fines were very less observed in 4% binder concentration than 2% binder concentration. Aggregation of pellets was also less in 4% binder concentration than 2% binder concentration. Assay of three different batches was found distributed because of different proportion of binder concentration in different batches; 2% binder concentration assay was observed less and in 6% binder concentration higher than required. But in 4% binder concentration, assay was nearer to required assay. From the P.S.D data and % assay data, it was concluded that 4% polymer used as a binder shows good drug entrapment and particle size of pellets.

### Film coating on drug-coated pellets

From the sieve analysis study of pellets [Table 3], batch no. P 18 (8%) and P 19 (8.5%) showed good pellets. In 7% and 7.5% concentration, polymer film coating on pellets was observed not proper and also plasticity was not observed in pellets. Most of the film-coated pellets have to retain on the # 30 and # 40 sieve with minimum fines. This criterion was only observed in batch no. P 18 (8%), P 19 (8.5%), and P 20 (9.0%). But in batch no. P 20 aggregation of pellets was observed in little amount and sticking of pellets occurred to the each other. So from appearance and sieve analysis study, it was concluded that batch no. P 18 (8%) and P 19 (8.5%) show good pellets in comparisons with other batches. % Assay of all batches was acceptable and came within the range, except batch P 16. % Assay results of all batches showed that all batches contain equivalent amount of ramipril within the range. Because of final formulation of pellets was tablet, dissolution of pellets was carried out by converting it into tablet dosage form. *In-vitro* dissolution data of tablet were compared with the release profile of innovator dosage form (marketed). From the dissolution data, batch no. P 16 (7%) and P 17 (7.5%) show very fast release of ramipril. This may be because of the rupture of pellets during compression and this rupture of pellets also may cause the stability problem of ramipril. While batch no. P 18 (8%) and P 19 (8.5%) show good and comparable results with the innovator dosage form. And batch no. P 20 (9.0%) showed little retardation of release than the reference product. So, batch no. P 19 (8.5%) showed good release and had greater F_2_ value than other batches.

Stability study of 8.5% polymer concentration film-coated pellets showed no degradation of ramipril and similar dissolution as initial at 40°C and 75% RH condition after 3 months. So, formulation of batch no. P 19, film-coated pellets was taken for the next optimization of tablet dosage form.

**Table 3:** Sieve analysis and assay and F_2_ value results of different % concentrations of film formers

ASTM sieve no.	%Weight retained of pellets				
B. No.	P-16 (7%Polymer)	P-17 (7.5%Polymer)	P-18 (8%Polymer)	P-19 (8.5%Polymer)	P-20 (9%Polymer)
20	0.7	0.6	1.8	5.8	10.1
30	42.8	50.8	55.1	66.1	71.8
40	35.8	30.1	30.7	24.3	15.8
60	12.2	7.6	4.8	1.3	1.4
80	2.5	4.1	4.1	0.7	0.2
100	3.3	3.8	2.2	1.1	0
Below 100	2.7	3	1.3	0.7	0.7
% Assay	105.1	103.3	101.8	99.8	98.0
F_2_ value for dissolution	61.40	66.49	76.62	81.36	60.09

### Tableting of coated pellets

These studies were performed to optimize the concentration or amount of diluents used in MCC (Avicel) and mannitol (Pearlitol SD 200) to give the plasticity to pellets and prevent the rupturing of pellets. In batch no. T1, only the MCC (Avicel) was used as a diluent (MCC 100%) and in batch no. T2 both diluents were used in equal amount (MCC-mannitol = 50-50%) and in batch no. T3, only mannitol (Pearlitol SD 200 – 100%) and was used as a diluent in tablet formulation [Table 4]. All the characteristics of these batches are discussed next.

**Table 4:** Accelerated stability study data of different %concentrations of film formers

Parameters	Initial	40°C 75%RH 3 M
% Assay (ramipril)	99.8	93.4
% Related impurities (total impurity)	BLOQ	3.63
% Dissolution (ramipril)	99.8	97.1

### Results of micromeritic properties of blend with different ratio of diluents

The micromeritic properties of different batches, i.e., bulk density, tapped density, compressibility index, Hausner ratio, and angle of repose, revealed no significant difference among the batches. Batch no. T1 shows the good flow, compressibility, and other parameters than other two batches. Loss on drying was also observed to be in the range of 2-3% wt/wt for all batches, which passes the USP requirements. Thus, from the micromeritic data it is evident that blends of different batches possess comparable compressibility and flow properties of all batches prepared with MCC and Pearlitol SD 200 [Table 5].

**Table 5:** Micromeritic properties of blend with different ratio of diluents (B. T1, T2, T3)

Batch code	B.D (g/cc)	T.D (g/cc)	C.I (%)	H.R	A.R (φ)	% LOD
T 1 (100%	0.64	0.72	12.82	1.15	26.50	2.45
MCC)						
T 2 (50/50%	0.63	0.73	13.79	1.16	24.10	2.61
MCC/mannitol)						
T 3 (100%	0.61	0.75	18.66	1.23	31.45	2.09
mannitol)						

### Physical characteristics of tablets

The tablets of different batches were prepared at same adjustment of machine; after that physical characteristics were found to be consistent as in all batches and shown in Tables 2, 6 and 5. All characteristics of tablets were within the limit, and acceptable and comparable with the reference product except the disintegration time and friability [Table 7].

**Table 6:** Sieve analysis and assay results of different % concentrations of binder

ASTM sieve no.	%Weight retained of pellets				
	2% Binder	4% Binder	6% Binder
20	0	0	0.86
30	0.2	2.3	8.94
40	41.7	73.8	71.9
60	32.2	20.4	14.2
80	15.4	1	2.1
100	3.8	0.7	0.8
Below 100	6.7	1.8	1.2
%Assay	94.1	99.6	106.8

**Table 7:** Physical characteristics of tablet with different ratio of diluents (B. T1, T2, T3)

Batch code	Avg. weight (mg)	Hardness (N)	Thickness (mm)	Friability (% w/w)	D.T. (min)
T 1 (100% MCC)	196.7–201.8	64–77	2.92–3.07	0.314 %	0.23
T 2 (50/50% MCC/mannitol)	197.8–202.1	51–60	3.01–3.19	0.395 %	1.26
T 3 (100% mannitol)	197.1–200.9	53–60	2.98–3.13	Capping	4.48

### *In vitro* dissolution study

**Figure 1:**
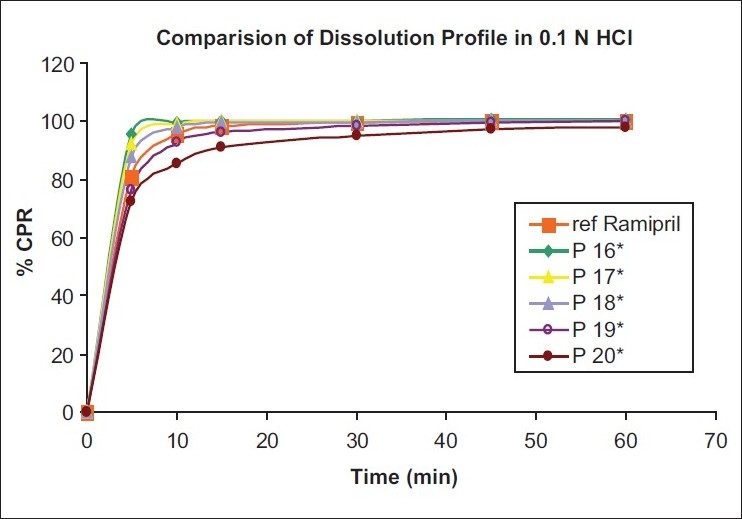
Dissolution profiling of different percentage concentrations of film formers

**Figure 2:**
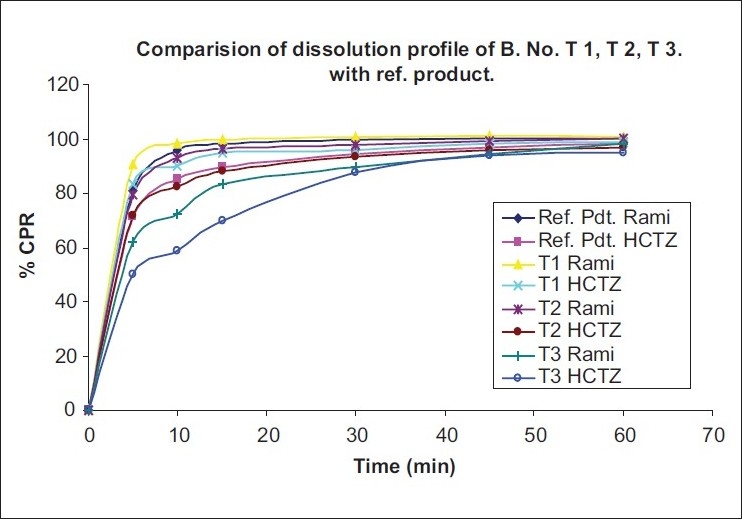
Dissolution profiling of different ratio of diluents (B. T1, T2, T3)

Tablets containing only MCC showed very fast dissolution into the medium. Those results were acceptable and also comparable with the reference product. Batch T2 showed the comparable and expected dissolution of tablets. But batch T3 showed delayed release of tablets than reference product release. In batch T1, fast release was observed because of only MCC also used as a disintegrating agent and an increase in the concentration of MCC, it will increase disintegration of tablets. In batch T3 only pearlitol SD 200 was used, which is crystalline, water-soluble diluents, and does not have any disintegration property. So, this batch showed delayed release due to long time for disintegration of tablets. But in batch T2, MCC and pearlitol SD 200 used in same concentration showed similar results to the reference product release. Similarity study of all batches is shown in Table 8, which shows that B. No. T3 has F_2_ value less than 50 (43.4% for ramipril and 41.1% for HCTZ) for both ramipril and HCTZ release compared with reference product release, so this batch was not acceptable. Batch no. T1 showed F_2_ value in the range of 50-100 (69.2% for ramipril and 63.1% for HCTZ) for both ramipril and HCTZ release, compared with reference product release. So, this batch was acceptable. But batch no.T2 had shown F2 value in the range of 50-100 and also nearer to 100 (87.0% for ramipril and 88.1% for HCTZ), which showed good similarity with reference product release of ramipril and HCTZ than batch no. T1. Dissolution profile is given in Figures 1 and 2.

**Table 8:** F2 value of tablet with different ratio of diluents (B. T1, T2, T3) with comparison to reference

Batch code	F2 value with comparison to reference	
	Ramipril (%)	HCTZ (%)
T 1 (100% MCC)	69.2	63.1
T 2 (50/50% MCC/mannitol)	87.0	88.1
T 3 (100% mannitol)	43.4	41.1

### Accelerated stability study

Accelerated stability data show that ramipril and hydrochlorothiazide drugs were not degraded after 3 months also. And all impurities were also found in acceptable range. After a 3-month accelerated stability study, there was no change in the tablet disintegration time and water content of the tablets. After 3 months, dissolution of tablets was performed and compared it with the initial dissolution of tablets. The results were not affected much during this 3-month period [Table 9].

**Table 9:** Accelerated stability study data of different ratio of diluents

Parameters	Initial	40°C 75% RH	30°C 75% RH
		3 M	3 M
% Assay (ramipril: HCTZ)	100.3/98.8	97.7/97.8	100.2/98.3
% Related impurities (total	BLOQ	2.35	1.49
impurity)			
% Dissolution (ramipril:	99.7/96.2	97.6/94.7	98.3/95.1
HCTZ) in 45 min			
% Water Content	3.89	6.63	6.62
Disintegration time (min)	1–17	1–37	1–29

## CONCLUSION

Formulation development of ramipril pellets for improving the stability from stress condition with required dissolution of tablets was tried with film coating, which showed a decrease in the degradation of the ramipril after formulation process. After a 3-month accelerated stability study of ramipril, pellets and MUPS have shown related impurities of 2.35% and 1.49%, respectively, which complied the limit of impurities (NMT 5.0%) required in final formulation.

MUPS prepared with super disintegrating agents showed 100.3% dissolution of ramipril and 98.8% dissolution of hydrochlorothiazide within 45 min. This showed that a better dissolution of MUPS was achieved within 45 min.

Whole investigation summarized that MUPS prepared by incorporating ramipril pellets and hydrochlorothiazide with other excipients showed good stability along with degradation of ramipril and immediate action of tablet with highest bioavailability of both drugs.

## References

[1] Heidbreder D, Froer KL, Bauer B, Cairns V, Breitstadt A, Bender N (1993). Combination of ramipril and hydrochlorothiazide in the treatment of mild to moderate hypertension- part 2: An open long term study of efficacy and safety. Clin Cardiol.

[2] Rang HP, Dale MM (1999). Pharmacology.

[3] Koytchev R, Ozalp Y, Erenmemisoglu A, van der Meer MJ, Alpan RS (2006). Effect of the combination of lisinopril and hydrochlorothiazide on the bioequivalence of tablet formulations. Arzneimittelforschung.

[4] (2006). Stabilized Individually Coated Ramipril Particles, Compositions and Methods, Wo2006050533, 11 May.

[5] (2006). Stabilized Ramipril compositions and methods of making, WO2006052968, 18 May.

[6] Jalal IM, Malinowski HJ, Smith WE (1972). Tablet granulations composed of spherical-shaped particles. J Pharm Sci.

[7] Parikh BM (1990). Alternatives for Processing Spherical Granules, paper presented at Interphex USA, 10 May.

[8] Bodmeier R (1997). Tableting of coated pellets. Eur J Pharm Biopharm.

[9] Celik M, Ghebre-Sellasie I (1994). Compaction of multiparticulate oral dosage forms. Multiparticulate oral drug delivery.

[10] Juslin M, Turakka L, Puumalainen P (1980). Controlled release tablets. Pharm Ind.

[11] Shafiq S, Shakeel F, Talegaonkar S, Ahmad FJ, Khar RK, Ali M Development and bioavailability assessment of Ramipril nanoemulsion formulation. Eur J Pharm Biopharm.

[12] Koytchev R, Ozalp Y, Erenmemisoglu A, van der Meer MJ, Alpan RS (2004). Effect of the combination of lisinopril and hydrochlorothiazide on the bioequivalence of tablet formulations. Arzneimittelforschung.

[13] Li CL, Martini LG, Ford JL, Roberts M (2005). The use of Hypromellose in oral drug delivery. J Pharm Pharmacol.

[14] (2006). Stabilized Ramipril compositions and methods of making, WO2006052968, 18 May.

[15] http://www.nbent.com/details.htm.

[16] Rowe RC, Sheskey PJ, Weller PJ (2003). Handbook of Pharmaceutical Exipients.

[17] Rowe RC, Sheskey PJ, Weller PJ (2003). Handbook of Pharmaceutical Exipients.

[18] Lowenthal W (1973). Mechanism of action of tablet disintegrants. Pharm Acta Helv.

[19] Smith G, Mclntosh IE (1976). Suspending agents for extemporaneous dispensing [letter]. Pharm J.

[20] Khan KA, Rhodes CT (1975). Water sorption properties of tablet disintegrants. J Pharm Sci.

[21] Rowe RC, Sheskey PJ, Weller PJ (2003). Handbook of Pharmaceutical Exipients.

[22] Shangraw R, Mitrevej A, Shah M (1980). A new era of tablet disintegrants. Pharm Technol.

[23] Rowe RC, Sheskey PJ, Weller PJ (2003). Handbook of Pharmaceutical Exipients.

[24] http://www.nbent.com/details.htm.

[25] http://www.en.wikipedia.org/wiki/microcrystalline_cellulose.

[26] Laicher A, Lorck CA, Tobin J, Stanilaus F (1994). Process optimization of pellet coating and drying using fluid-bed production units. Pharm Tech Eur.

[27] El-Mahrouk GM, Al-Meshal MA, Al-Anagary AA, Mahrous GM (1993). Preparation and evaluation of sustained-release indomethacin nonpareil seeds. Drug Dev Ind Pharm.

[28] Hosny EA, El-Mahrouk GM, Gouda MW (1998). Formulation and *in vitro* and *in vivo* availability of diclofenac sodium enteric-coated beads. Drug Dev Ind Pharm.

[29] (1996). Ministry of Health and Family Welfare. Indian Pharmacopoeia.

[30] (2000). British Pharmacopoeia.

[31] Lachman L, Lieberman A, Kinig JL (1991). The Theory and Practice of Industrial Pharmacy.

[32] Tang Y, Gan K (1998). Statistical evaluation of *in vitro* dissolution of different brands of ciprofloxacin hydrochloride tablets and capsules. Drug Dev Ind Pharm.

